# Modeling spatio-temporal effects of propofol using a neural field approach

**DOI:** 10.1186/1471-2202-14-S1-P29

**Published:** 2013-07-08

**Authors:** Manh Nguyen Trong, Thomas R Knösche, Ingo Bojak

**Affiliations:** 1 Max Planck Institute for Human Cognitive and Brain Sciences, 04103 Leipzig, Germany; 2 Institute for Biomedical Engineering & Informatics, Ilmenau University of Technology, 98693 Ilmenau, Germany; 3 School of Systems Engineering, University of Reading, Whiteknights, Berkshire, RG6 6AY, UK; 4 School of Psychology (CN-CR), University of Birmingham, Edgbaston, Birmingham, B15 2TT, UK

## Introduction

Anesthetic agents like propofol induce global changes of brain states and behavior [1]. Yet the relation to the observed spatio-temporal patterns of EEG and fMRI has not been fully understood. In this study, we use a biologically inspired neural field model to explain these patterns in the human brain induced by propofol.

## Methods

Each grey matter voxel in a T1-weighted MRI is modeled by a local circuit of neural masses as proposed by Jansen and Rit [2]. We then define two types of connections between them: local connections (W_L_) estimated by neural morphological properties [3] and (b) distal connections (W_D_) characterized using diffusion weighted MRI. The entire model is described by a system of integral differential equation:

$$\text{Θ}\left\{\left(V\left(r,t\right)\right\}=HT\right)\underset{\text{Ω}}{\int }W_{L}\left(r,\tilde{r}\right)S\left[V\left(r,t\right)\right]d\tilde{r}+HT\underset{\text{Ω}}{\int }W_{D}\left(r,\tilde{r}\right)S\left[V\left(r,t-t^{\left(d\right)}\right)\right]d\tilde{r}+I\left(r,t\right)$$

where Θ is the differential operator accounting for the synaptic-dendritic dynamics, **V **is the vector of membrane potentials, **S **is a sigmoid function, t^(d) ^is the time delay due to distal connections, **H**, **T **are the synaptic gains and characteristic time constants and **I **is the external input. With an appropriate neurovascular coupling and Balloon-Windkessel hemodynamic, the simulated ECoG (25 minutes) can be converted into changes of fMRI BOLD.

Results

**Figure 1:**
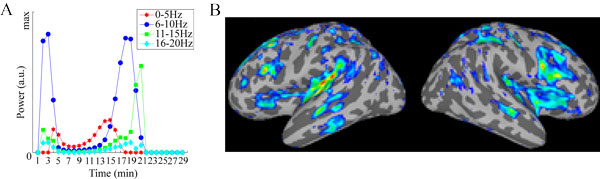
**Simulated cortical activity**. A: Temporal characteristics of various frequency bands. B: Spatial distribution of power.

**Figure 2:**
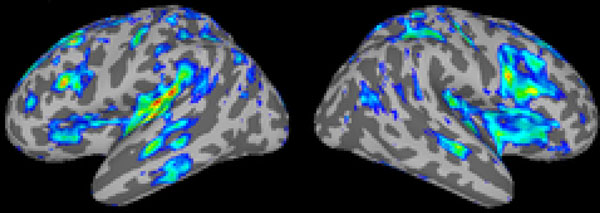


Simulation results (see Figure 1) match experimental EEG data from [1], which shows biphasic peaks in the power in all investigated frequencies during the loss and return of consciousness. The simulated distribution of fMRI BOLD predicts strong activity in the left temporal lobe and bilateral middle temporal sulci (see Figure 2).

## Conclusions

Our realistic model of the whole brain provides a powerful computational tool for investigating the spatio-temporal EEG and fMRI BOLD patterns during the application of propofol. Preliminary results match well both spatial and temporal characteristics of the experimental data.
